# Improved Feature Point Pair Purification Algorithm Based on SIFT During Endoscope Image Stitching

**DOI:** 10.3389/fnbot.2022.840594

**Published:** 2022-02-15

**Authors:** Yan Liu, Jiawei Tian, Rongrong Hu, Bo Yang, Shan Liu, Lirong Yin, Wenfeng Zheng

**Affiliations:** ^1^School of Automation, University of Electronic Science and Technology of China, Chengdu, China; ^2^Department of Geography and Anthropology, Louisiana State University, Baton Rouge, LA, United States

**Keywords:** endoscope, feature point matching, image mosaic, SIFT algorithm, K-nearest, RANSAC

## Abstract

Endoscopic imaging plays a very important role in the diagnosis and treatment of lesions. However, the imaging range of endoscopes is small, which may affect the doctors' judgment on the scope and details of lesions. Image mosaic technology can solve the problem well. In this paper, an improved feature-point pair purification algorithm based on SIFT (Scale invariant feature transform) is proposed. Firstly, the K-nearest neighbor-based feature point matching algorithm is used for rough matching. Then RANSAC (Random Sample Consensus) method is used for robustness tests to eliminate mismatched point pairs. The mismatching rate is greatly reduced by combining the two methods. Then, the image transformation matrix is estimated, and the image is determined. The seamless mosaic of endoscopic images is completed by matching the relationship. Finally, the proposed algorithm is verified by real endoscopic image and has a good effect.

## Introduction

Endoscopy is one of the most commonly used detection tools in clinical practice (Rosen and Ponsky, 2001; Zhou et al., 2016; Yang et al., 2017; Ni et al., 2019). It plays a very important role in determining and treating diseases. However, the result of detection would depend on the amount of information delivered by the endoscopic imaging (Liu et al., 2015, 2018), which have been discussed in many other disciplines (Zheng et al., 2015, 2016a; Li et al., 2017, 2020; Yin et al., 2019; Tang et al., 2020b). It is impossible to get the best field of view and magnification of an endoscopic image at the same time. For example, the larger the image's magnification, the more detailed the image information will be. However, the field of vision information contained in the image will become smaller. Therefore, in the case of large magnification, it is impossible to realize the inspection of large organs at one time. This has a great influence on doctors in judging the details and scope of the disease in detail. However, the qualitative judgment of the disease is not helpful (Tang et al., 2020a).

Early endoscopic image stitching techniques mostly used a combination of frequency domain correlation algorithms (Ellmauthaler et al., 2012; Chen and Dai, 2013; Li et al., 2015; Tang et al., 2020a) and maximum mutual information (Zheng et al., 2016b, 2017; Yang et al., 2018; Chen et al., 2020; Xu et al., 2020). For example, Mier et al. (2006) proposed an automatic stitching algorithm for two-dimensional cryptoscopic sequence images. The algorithm is robust to fuzzy, illumination, and heterogeneous radial distortion images. And it can use the cancer autofluorescence effect in the image to detect cancer lesion information. In 2004, Lowe (2004) proposed SIFT based on scale invariance, which is an algorithm to describe local features. The main approach is to construct Gaussian pyramids of different scales for images. The feature points to be obtained are the extreme values detected in the difference pyramid. The algorithm has great robustness in the case of image viewing angle, scale change or rotation, and it has a certain milestone in feature extraction. In response to the complexity of SIFT calculations, the SURF (speed up robust features) feature (Bay et al., 2006), binary SIFT feature (Peker, 2011), and the GLOH (gradient location orientation histogram) feature (Mikolajczyk and Schmid, 2005) were developed. After that, the researchers applied the SIFT feature detection algorithm to endoscopic image mosaic technology. Behrens (Behrens, 2008) proposed a two-dimensional homographic matrix based on the SIFT feature point estimation image. It used an endoscopic bladder fluorescence image stitching algorithm that combines an affine model and an adaptive iterative algorithm. The algorithm has a good splicing effect. However, the amount of calculation is relatively large. Therefore, he improved the algorithm afterwards (Behrens et al., 2009), and solved the problems of waveform correction of images after cystoscope splicing and automatic recognition of sequence image space. Burkhardt et al. (2013) proposed a microrobot with flexible motion and an automatic bladder scanning system. The robot can adjust the endoscope's motion based on the image's feedback information to capture the image of the bladder surface. Then the image is matched based on SIFT feature points to realize automatic image stitching. Robot-assisted surgery can completely realize the unsupervised state (Behrens et al., 2009).

Chen et al. (2010) proposed an endoscopic image mosaic algorithm based on the large intestine. The algorithm uses SURF for feature matching and simplifies the dimension of features and the main direction of calculation features. It improves the speed of splicing. The effect of stitching is also very good, which is more suitable for endoscopic image mosaic. Rosten et al. (2008) proposed a FAST algorithm for corner detection. This algorithm is based on SUSAN (small univalve segment assimilating nucleus) corner detection. In the search process, the speed of feature point detection is greatly improved. However, the disadvantage of FAST is that it does not have scale invariance. Therefore, Rublee et al. (2011) proposed a new method based on FAST and BRIEF (binary robust independent element feature). Based on this algorithm, a fast matching algorithm ORB (Oriented Brief) is proposed to solve the problem of susceptibility to noise and lack of scale and deformation. In recent years, because of their excellent quality and development of the deep learning, binary descriptors are popular in keypoint detection and registration and are widely used in image alignment. Especially recently, there are more and more experts and scholars studying local binary image feature descriptors, which makes the binary description develop very rapidly. Many new binary descriptors, such as BRISK (Leutenegger et al., 2011) (binary robust invariant scalable keypoints) and FREAK (fast retina keypoint) (Alahi et al., 2012), have been produced. Compared with the ORB, fixed sampling mode is used to replace random sampling structure mode. Although the ORB algorithm has been used in many image mosaics, it is still seldom used in endoscopic image mosaic. For example, Wang et al. (2004) and others proposed a method of aerial image mosaics using ORB features. The matching feature in this algorithm is to use ORB feature points and use binary feature vectors to calculate the distance of feature points. Therefore, the speed of feature extraction and matching has been greatly improved. In the image matching, cross-validation algorithm, next nearest neighbor screening algorithm, and RANSAC estimation algorithm are used to calculate the homography matrix between the sequence images to complete the mosaic.

Similarly, endoscopic instruments have been further improved with the advancement of technology. Luo et al. (2021) proposed a human motion intention prediction method based on an autoregressive (AR) model for teleoperation. The proposed human motion prediction algorithm acts as a feedforward model to update the robot's motion and to revise this motion in the process of human-robot interaction (HRI). Then, Su et al. (2022) applied the swivel motion reconstruction approach to imitate human-like behavior using the kinematic mapping in robot redundancy. They proposed a novel incremental learning framework that combines an incremental learning approach with a deep convolutional neural network for fast and efficient learning.

Although there are many stitching algorithms in image mosaic technology, these algorithms are generally suitable for ordinary images. In this paper, an improved stitching algorithm is proposed for endoscope images. This paper proposes to combine the nearest neighbor matching method with RANSAC (random sample consensus) matching algorithm. Firstly, the distance ratio between the nearest neighbor and the next nearest neighbor is used to determine the matching point pairs preliminarily. Secondly, the RANSAC matching algorithm eliminates the mismatched points and obtains the holography matrix between the corresponding frames. Then, the endoscopic image registration is performed according to the holography matrix. Finally, a weighted fusion algorithm based on gradual in and gradual out is used to fuse the registered images to eliminate the obvious gaps in image mosaic and realize a seamless mosaic of endoscopic panoramic images. The experimental results show that the mosaic effect is good, and the accuracy of feature point matching is improved.

## Method

In general, endoscope Mosaic technology consists of four parts: preprocessing image, matching image, image transformation and image fusion. It is necessary to analyze two or more endoscope images completely because the splicing of multiple continuous endoscopes is the problem to be solved by endoscope splicing technology. In this way, compared with image interpolation or compression and other processing technologies, the diversity, complexity and pertinence of endoscope Mosaic technology are different.

**Figure 1:**
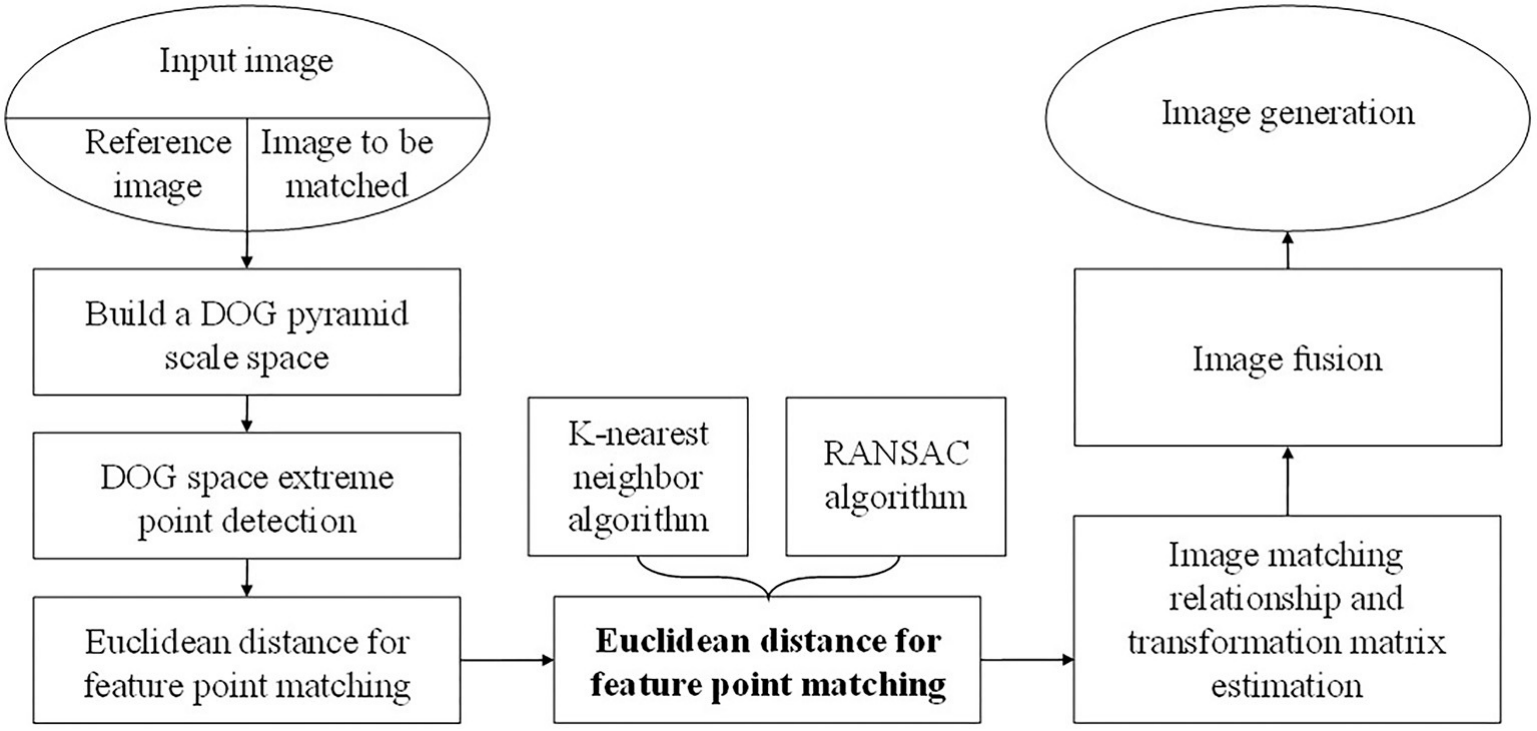
Flow chart of improved SIFT algorithm.

If there are many mismatches in the process of feature point matching, it will cause a great deviation in the image stitching results. However, the existing mismatch elimination technology generally only selects a single method, which has a low efficiency. To improve the efficiency, this paper proposes an improved feature point pair purification algorithm. First, use the keypoint matching algorithm based on K-nearest neighbors, that is, two-way registration to initially determine the matching point pairs, then use RANSAC to delete the wrong matching points and obtain the homography matrix between the corresponding frames. Finally, the registration of the endoscopic image is performed according to the homography matrix. The flowchart of the improved SIFT algorithm is shown in Figure 1.

### Principle of SIFT Algorithm

#### DOG Space Extreme Point Detection

The SIFT algorithm performs keypoint detection in a multi-scale space, and the Gaussian kernel is the only linear kernel that can complete the scale transformation. We take the convolution of the Gaussian kernel function *G*(*x, y*, σ) and the image *I*(*x, y*) as the scale space *L*(*x, y*, σ) of the image, that is, the LOG (Laplacian of Gaussian) operator:

(1) $$\begin{array}{l} L\left(x,y,\sigma \right)=G\left(x,y,\sigma \right)*I\left(x,y\right) \end{array}$$

*G*(*x, y*, σ) is a scale-variable Gaussian function.

(2) $$\begin{array}{l} G\left(x,y,\sigma \right)=\frac{1}{2\pi \sigma^{2}}e^{\frac{-\left(x^{2}+y^{2}\right)}{2\sigma^{2}}} \end{array}$$

Convolution is represented by the symbol ∗, the scale factor is σ, and each pixel position of an image is (*x, y*). Large scale is used if we need to know the general features of the image, whereas small scale is used if we need to know the detailed features of the image. This is because the larger the scale, the more the image is filtered.

In order to make the key points of detection more stable, David. Lowe et al. proposed to convolved the image with Gaussian difference function to obtain the extreme value of the scale space, i.e.,

(3) $$\begin{array}{l} D\left(x,y,\sigma \right)=\left(G\left(x,y,k\sigma \right)-G\left(x,y,\sigma \right)\right)*I\left(x,y\right)=L\left(x,y,k\sigma \right)\\ \;-L\left(x,y,\sigma \right) \end{array}$$

Where *k* represents the multiple relationship in adjacent images, and $k=\sqrt{2}$ is taken in this paper.

We usually use the response value of the DOG operator to approximate the value of the σ^2^∇^2^*G* operator, because the Gaussian difference operator can be equivalent to the Laplace operator to some extent.

Where, the relationship between *D*(*x, y*, σ) and σ^2^∇^2^*G* can be calculated as follows:

(4) $$\begin{array}{l} \frac{\partial G}{\partial \sigma }=\sigma \nabla^{2}G \end{array}$$

Calculate the difference of Equation (4) as follows:

(5) $$\begin{array}{l} \sigma \nabla^{2}G=\frac{\partial G}{\partial \sigma }\approx \frac{G\left(x,y,k\sigma \right)-G\left(x,y,\sigma \right)}{k\sigma -\sigma } \end{array}$$

That is:

(6) $$\begin{array}{l} G\left(x,y,k\sigma \right)-G\left(x,y,\sigma \right)\approx \left(k-1\right)\sigma^{2}\nabla^{2}G \end{array}$$

*D*(*x, y*, σ) can be approximated as σ^2^∇^2^*G*, because the constant (*k*−1) on the right side of the equation in Formula (3–6) generally does not interfere with the position of the key point. Therefore, this also proves that the DOG operator can be approximated as the LOG operator.

The advantages of the DOG operator are self-evident. (1) Since the information (size and scale) of each layer of images generated in the pyramid is contained in the DOG operator, we can directly use the spatial scale images obtained in Formula (1−3) and obtain the features in the images we need without recalculating the scale again; (2) The LOG operator takes less time to compute Gaussian convolution kernels. This is because compared with the LOG operator, DOG operator only uses one convolution kernels to compute. (3) Since DOG operator can be simplified and approximate to LOG operator, DOG has excellent properties of LOG. For example, LOG operator has invariability on noise, and it will be more stable than other detection methods when carrying out characteristic point detection, such as DoH of Hessian, Harris characteristic point detection, etc.

The strong edge response of DOG operator is caused by the weak response of some feature points, so the key points obtained are not necessarily stable. We can generally use a three-dimensional quadratic function to accurately determine the scale and location information of key points, so that those unstable and low contrast key points are also removed, so that the stability of registration is improved, and the anti-noise performance is also strengthened.

For the difference function *D*(*x, y*, σ), expand the second order of Taylor's formula:

(7) $$\begin{array}{l} D\left(x\right)=D+\frac{\partial D^{T}}{\partial x}x+\frac{1}{2}x^{T}\frac{\partial^{2}D}{\partial x^{2}}x \end{array}$$

Assume the extreme point $\hat{x}\;$ of *x* and assume that Equation (7) can be derived and the left-hand side of the equation should be 0.

(8) $$\begin{array}{l} \hat{x}=-\frac{\partial^{2}D^{-1}}{\partial x^{2}}\frac{\partial D}{\partial x} \end{array}$$

By substituting the values obtained in Formula (8) back into Formula (7), feature points with low contrast can be removed:

(9) $$\begin{array}{l} D\left(\hat{x}\right)=D+\frac{1}{2}\frac{\partial D^{T}}{\partial x}\hat{x} \end{array}$$

If the displacement of $\hat{x}$ in all directions is >0.5 relative to the interpolation center point, this point needs to be eliminated because the center point may have shifted to a nearby point. And when $|D\left(\hat{x}\right)|<0.03$, the response value point also needs to be deleted because it is too small so it is not stable when disturbed by noise.

Because the image edge will have a great influence on the stability of key points, if some key points are close to or even on the image boundary, then these points cannot be used in image registration because they are extremely unstable. Therefore, in order to prevent the detected key points from being affected by noise, it is not enough to delete only the points with low contrast. Since points on the boundary are easily unstable when affected by noise, and it is difficult for us to accurately determine the position of points on the boundary, it is necessary to delete key points on the boundary.

Strong edge response is the shortcoming that DOG operator cannot overcome. In order to determine whether some key points are at the image boundary, we can use the principal curvature to judge. Hessian matrix *H* is a method to calculate the magnitude of the principal curvature:

(10) $$\begin{array}{l} H=\left[\begin{array}{cc} D_{xx} & D_{xy}\\ D_{xy} & D_{yy} \end{array}\right] \end{array}$$

Among them, we only need to find the ratio between the eigenvalues and eigenvalues in the *H* matrix instead of figuring out the eigenvalues one by one. The value of D can be obtained by calculating the difference of the gray values of the surrounding points. If we set the minimum eigenvalue of H as β = λ_min_ and the maximum eigenvalue as α = λ_max_, then the magnitude and determinant of the trace of H can be expressed by α and β :

(11) $$\begin{array}{l} \{\begin{array}{c} Tr\left(H\right)=D_{xx}+D_{yy}=\alpha +\beta \\ Det\left(H\right)=D_{xx}D_{yy}-{\left(D_{yy}\right)}^{2}=\alpha \beta \end{array} \end{array}$$

Let α = γβ, α is represented by β and proportional coefficient γ :

(12) $$\begin{array}{l} \frac{Tr{\left(H\right)}^{2}}{Det\left(H\right)}=\frac{{\left(\alpha +\beta \right)}^{2}}{\alpha \beta }=\frac{{\left(\gamma \beta +\beta \right)}^{2}}{\gamma \beta^{2}}=\frac{{\left(\gamma +1\right)}^{2}}{\gamma } \end{array}$$

From Equation (12), we can see that the equation depends only on the ratio of α to β and not on their size. $\frac{{\left(\gamma +1\right)}^{2}}{\gamma }$ decreases as the ratio of α to β decreases. To minimize $\frac{{\left(\gamma +1\right)}^{2}}{\gamma }$, all you need is alpha and beta to be equal. Therefore, we can determine whether the principal curvature is less than the threshold γ through Equation (13).

(13) $$\begin{array}{l} \frac{Tr{\left(H\right)}^{2}}{Det\left(H\right)}<\frac{{\left(\gamma +1\right)}^{2}}{\gamma } \end{array}$$

It works best when γ = 10. That is, we keep the key points where the ratio of alpha to beta is <10, and eliminate the ones that don't meet the requirement.

#### SIFT Feature Point Description

When describing the key points, it is necessary to assign a main direction to them one by one. The main purpose is to realize the invariance of image rotation, and the main idea is based on the gradient direction and magnitude nature of amplitude. Difference is used to solve the magnitude and direction of the gradient in a circle with a radius of 3 × 1.5σ at the center of the key point:

(14) $$\begin{array}{l} \{\begin{array}{ll} m(x,y) & =\sqrt{{\left(L(x+1,y)-L(x-1,y)\right)}^{2}+{\left(L(x,y+1)-L(x,y-1)\right)}^{2}}\\ \theta (x,y) & =\frac{\tan^{-1}((L(x,y+1)-L(x,y-1))}{L(x+1,y)-L(x-1,y)}) \end{array} \end{array}$$

The sampling is centered on feature points, and histogram is used to describe the gradient and direction. L represents the scale of feature points. The CVD has 36 CVD directions in the histogram, so it ranges from 0 to 360°. It is necessary to use gaussian function to weight the magnitude of the modulus of the gradient when calculating the direction of the histogram.

The direction of the gradient around the key point is represented by the peak value of the histogram. In general, the main direction of the key point is the highest in the histogram, while the direction around the key point is distributed at the rest of the peak value. In order to enhance the robustness of matching, usually there will be multiple auxiliary directions of SIFT feature points, that is, when they are >80% of the peak value, in order to make them have better robustness in image alignment. Although only 15% of these key points may have multiple auxiliary directions, the stability of key point registration has been greatly improved.

Each key point contains three kinds of information: direction, scale, and location. The key points are described so that they are stable without interference from external factors, so that they are invariant to changes in Angle or light. The uniqueness of the descriptor is important in order to achieve higher accuracy in key point matching. Generally, in order to make the key point in a more appropriate scale, it is necessary to sample the surrounding pixels, and then use the normalized correlation algorithm to match the pixel gray. However, simple correlation is very sensitive to affine, non-rigid deformation and 3D perspective changes, which can lead to mismatching of samples.

Feature vectors can be obtained through the structural order of key points. And the SIFT descriptor is a vector that has a lot of dimensions because it computes the gradient histogram. Descriptors need to be computed at the image scale because it is directly related to the scale size. The neighborhood of the key point is divided into 4 × 4 areas with side length of 3σ, i.e., 16 small areas. σ represents the scale size. The key point's neighborhood needs to expand to $15\sqrt{2}\sigma$ because it needs to be interpolated. We get the critical neighborhood and finally $15\sqrt{2}\sigma$ because there's a certain amount of rotation.

In order to make the key point invariant to light changes, the feature vectors need to be uniformly processed, that is, divided into the interval [0, 1]. When unifying key points, the values below 0.2 remain unchanged, and those above 0.2 are fixed as 0.2. In this way, key points are unique. Therefore, SIFT operator has good robustness for noise interference and affine changes, with invariance for image scale, light rotation changes.

In the case that the key point is centered, the direction of the pixel gradient in the neighborhood of the key point is rotated by an Angle θ. The main purpose is to make it invariant to rotation change. Rotate the key point neighborhood to the main direction and divide it into 4 × 4 small blocks of 3 sigma. Compute eight directions in each small block, then sum the results of the directions and get the seed points. The gradient histogram is divided by each sub-block into eight directions with a size of 45°, which is different from the calculation of the main direction of the key point at this point, so the gradient information of the seed point has eight different directions. Since there is a seed point containing eight directional gradients in each of the 16 sub-blocks, we can obtain the SIFT feature vectors with a total dimension of 16 × 8 = 128, and use a Gaussian function with a variance of 6σ to obtain the 128-dimensional feature vectors.

### Feature Matching

The main idea of feature matching is to use distance function to judge the similarity of feature description vector. Experts and scholars have done a lot of research work in the search for matching points, and many new algorithms have been proposed. However, there is no algorithm that can detect all the registration points of key points in multi-dimensional space. Because this article gets a descriptor is a higher dimension feature vector, use of Kd-Tree search algorithm is one of the best algorithms, however, when more than 10 d Kd-Tree search algorithm performance is poorer, therefore the BBF search algorithm based on the approximation algorithm, this method improves the search time, and you can find more matching points. In a word, BBF is an optimization algorithm.

#### Kd-Tree Search Algorithm

Kd-Tree is a binary Tree structure. Kd-Tree algorithm has many advantages: (1) since the segmentation superplane will keep changing in the process of building KD tree, data points of different clusters can be easily distinguished. (2) Since the resolution of the image can be adjusted when cutting the KD tree space, the height of the tree can be adjusted through the distribution of data points. (3) The cutting surface of KD tree can be adjusted according to different situations. In the process of building KD tree, a data point can be calculated by using the partition hypersurface equation, and the point can be judged to be in the right subtree or the left subtree.

#### Best Bin First Algorithm

The core of BBF algorithm is to add the search priority part, the rest of the process is similar to the standard KD tree. BBF algorithm is a kind of nearest neighbor optimization algorithm, which gives priority to the points with high matching possibility. Either when all the nodes in the priority queue have been searched or when the preset time runs out, it treats the optimal point of the search as the registration point because of its timeout setting. BBF algorithm can solve the problem that the nearest neighbor cannot handle multidimensional vector after extending KD tree. As a result, the speed of BBF searches is increased, but the accuracy is greatly reduced. Therefore, the results obtained by BBF are relatively good but not the best.

You can see the appropriate benefit of BBF for procedural manipulation of query best-points. The search process can be interrupted at any time through the construction of the priority queue, which is very widely used in high-dimensional data search due to its good results.

Because BBF spends a lot of time searching the nodes of the priority queue, the number of nodes must be manipulated properly. As the number of nodes increases, the search time increases, so the purpose of setting the number of nodes to 200 in this article is to control the search time. In addition, the order of searching leaf nodes depends on the structure of KD tree, so the location of searching node may be ignored even if the location of node storage is known. This problem can be solved by using the distance between the node being searched and the current node to query the node. If the Euclidean distance between the target point and the query point is smaller than the distance between the target point and the nearest matching point, then the nearest matching point is the query point.

### Improved Feature Point Pair Purification Algorithm

To improve the efficiency, this paper proposes an improved feature point pair purification algorithm. First, use the keypoint matching algorithm based on K-nearest neighbors, that is, two-way registration to initially determine the matching point pairs, then use RANSAC to delete the wrong matching points and obtain the homography matrix between the corresponding frames.

#### Feature Point Matching Algorithm Based on K-Nearest Neighbor

Before using RANSAC to remove the external points to calculate the transformation matrix parameters, a preliminary selection is made through the distance ratio between the nearest neighbor and the next nearest neighbor of the keypoint. This can reduce the workload of RANSAC and improve the purity of the matching point pairs using RANSAC. The K-nearest neighbor algorithm used in this paper can avoid large amounts of calculation caused by the exhaustive search method. The *K* value is taken as 2. Finally, each keypoint will get two matching points with the smallest and the next smallest distance. Let the eigenvector of the key points *p* be *V*_*p*_, the vector of the closest matching point *q* in the first image is *V*_*q*_ and the vector of the next closest matching point $\bar{q}$ in the first image is $V_{\bar{q}}$, then the description matching $p_{i}\left(V_{q},V_{\bar{q}}\right),i=1,2,\cdots \;$ can be used. *n* is the number of key points extracted from the second image.

Assuming $V_{\bar{q}}$ that are the adjacent key points $V_{\bar{q}}$ in the first image. To make *p* and *q* become a set of accurate registration pairs, *R* needs to be smaller than the critical value *T*_*R*_. At this time, it is represented by *p* ↔ *q*, and vice versa *p* ← | → *q*. Then the distance ratio is expressed as follows.

(15) $$\begin{array}{l} R=\frac{D\left(V_{p},V_{q}\right)}{D\left(V_{p},V_{\bar{q}}\right)} \end{array}$$

(16) $$\begin{array}{l} V_{q}=argmin\left\{D\left(V_{p},V_{i}\right)|V_{i}\in B,i=1,\cdots \;,m\right\} \end{array}$$

(17) $$\begin{array}{l} V_{\bar{q}}=argmin\left\{D\left(V_{p},V_{j}\right)|V_{j}\in B,j=1,\cdots \;,m,V_{j}\neq V_{q}\right\} \end{array}$$

(18) $$\begin{array}{l} \{\begin{array}{cc} p↔q & \;if\;R<T_{R}\\ p\leftarrow |\to q & otherwise \end{array} \end{array}$$

The experimental results show that when the critical value *T*_*R*_ of Equation (18) is 0.65, the accuracy and the matching logarithm of keypoints can get better results. If the set *A* is used to represent the final registration pair, then, $A=\left\{p_{i}\left(V_{q},V_{\bar{q}}\right)|i=1,2,\ldots m_{1}\right\}$. *m*_1_ is the number of key point registration pairs in the corresponding second frame of the first frame.

Then, in the second frame image, the nearest and next closest registration points of all the key points matching in the first frame image are searched. According to the above process, the error points whose *R* larger than the threshold value *T*_*R*_ are eliminated. The final keypoint registration pair is represented by a set *B*, that is, $B=\left\{q_{i}\left(V_{p},V_{\bar{p}}\right)|i=1,2,\cdots m_{2}\right\}$, *m*_2_ is the number of key point registration pairs in the corresponding first frame of the second frame image.

Finally, all the data are searched. For a certain registration pair (*p*_*i*_, *q*_*j*_) in the set *A*, if a matching pair (*q*_*j*_, *p*_*i*_) can be found in the set *B*, this point is accepted. The set *C* is used to store the keypoint pairs obtained through bidirectional registration, where $C=\left\{p_{i}\left(V_{q},V_{\bar{q}}\right)|i=1,2,\cdots m_{3}\right\}$. *m*_3_ is the number of keypoint pairs obtained through bidirectional registration.

#### RANSAC Algorithm

RANSAC is a robust parameter estimation algorithm (Triggs et al., 1999) proposed by Fischler and Bones. At first, people used this algorithm to estimate camera motion, and now it is widely used in parameter estimation. RANSAC can accurately get the correct registration relationship and eliminate the wrong matching points.

Firstly, the ratio of the nearest neighbor and the next nearest neighbor is used to express the registration results. The purpose of holography matrix detection is to find the most matching key points and eliminate those that do not meet the requirements. Therefore, we have the initial set of matching points.

For further purification of the key points after two-way registration, RANSAC's main approach is to assume a model applicable to all the key points that are correctly registered. The assumed interior points can be calculated from all the unknown parameters. All the data points are randomly sampled, and some points are set as interior points to form a random subset. Finally, random sampling is carried out until we find a transformation parameter model that can make the number of interior points maximum. Therefore, we need to remove the interference caused by mismatching points when we get interior points.

Generally, the transformation model with eight parameters is mainly used in splicing, the matrix transformation with 8 degrees of freedom. Because a pair of registration points can support two equations to establish the relationship, four non-collinear matching pairs are needed to calculate the parameters (Zhang et al., 2021).

The steps of RANSAC are as follows:

Four pairs are randomly selected in the registration set to solve the transformation matrix.

In solving the remaining registration pairs' matrix transformation, we need to use the transformation parameters and calculate the coordinate distance between points and point pairs. The inner point is the point within the error range, and the other points are called the outer point. In the first random sampling process, the maximum number of interior points is *X*, and we set *MAX* as *MAX* = *X* in the first arbitrary sampling process. If the ratio *X* is larger than *MAX*, then *MAX* = *X*.

This process is continued until the number of interior points is no longer increased. And the point set larger than the preset critical value of the number of interior points is regarded as the largest interior point set. The matrix parameter model is solved by using the least square method.

It is also necessary to set the maximum number of sampling, the critical value of distance, and the minimum number of points.

1. The determination of the maximum sampling times.
  If all the subsets are listed, it will lead to excessive sampling and a heavy workload. Therefore, we need to set an appropriate sampling number to ensure that the probability of any selected four pairs of registration points to the subset is large enough. Let the probability that all sampling points are interior points is *p*. In order to ensure the accuracy of the calculation, we make *p* as 99%. If the probability of the registration point belonging to the interior point is *p*_1_, then the probability of the registration point belonging to the outer point ε = 1 − *p*_1_. When calculating the matrix parameters, let the minimum registration point pair be *m*. In case of *N* samplings:
  (19) $$\begin{array}{l} {\left(1-p_{1}^{m}\right)}^{N}=1-p \end{array}$$
  Therefore, the sampling times are as follows:
  (20) $$\begin{array}{l} N=\frac{\log\left(1-p\right)}{\log\left(1-{\left(1-\varepsilon \right)}^{m}\right)} \end{array}$$
2. The determination of the critical value of the distance.
  If the error of registration points satisfies a Gaussian function, the standard deviation and mean value are 0. After the projection transformation, the sum of the square of the distance between the keypoints and the registration points is $d_{v}^{2}=d\left({x_{i}}^{{}^{\prime }},Hx_{i}\right)$. We set the significance *m* = 0.01 and the $\chi_{m}^{2}$ confidence level is 0.99.
  (21) $$\begin{array}{l} F_{m}\left(k^{2}\right)=\underset{0}{\overset{k^{2}}{\int }}\chi_{m}^{2}\left(\xi \right)d\xi <k^{2} \end{array}$$
  So, the critical distance is
  (22) $$\begin{array}{l} t^{2}=F_{m}^{-1}\left(\alpha \right)\sigma^{2} \end{array}$$
  σ^2^ is the variance of the Gaussian function.
  The following formulas are the judgment conditions of the inner point and the outer point, respectively:
  (23) $$\begin{array}{l} \{\begin{array}{cc} inner\;point & d_{v}^{2}<t^{2}\\ exterior\;point & d_{v}^{2}\geq t^{2} \end{array} \end{array}$$
3. The determination of the minimum number of interior points.

If there are n matching pairs in the registration, we can get the number of interior points is *n*(1 − ε). Generally, the number of interior points will not change after some iterations.

### Image Matching Relation and Transform Matrix Estimation

In the process of image registration, there may be no overlap, or there may be overlap. These situations will lead to an incorrect registration relationship. The stitching error caused by this situation will not be reminded, so whether the transformation matrix between images is correct is crucial to the success of stitching. However, the parameter model is not correct, which will lead to an incorrect registration relationship. Therefore, whether the transformation matrix between images is correct or not is crucial to stitching success. Therefore, a probability model is proposed to distinguish whether the registration is correct or not.

In the process of image matching, *n*_*f*_ represents the number of initial registration pairs before purification. *n*_*i*_ represents the number of interior points after eliminating the error points. By mathematical deduction, we can get that the number of interior points satisfies a binomial distribution.

*p*_1_ represents the probability that the registration point is an interior point under the condition of correct registration. *p*_0_ represents the probability that the registration point is an interior point under the condition of wrong registration.

(24) $$\begin{array}{l} P\left(f^{\left(1:n_{f}\right)}|m=1\right)=B\left(n_{i},n_{f},p_{1}\right) \end{array}$$

(25) $$\begin{array}{l} P\left(f^{\left(1:n_{f}\right)}|m=0\right)=B\left(n_{i},n_{f},p_{0}\right) \end{array}$$

The number of internal points $n_{i}=\sum_{i=1}^{n_{f}}f^{\left(i\right)}$. $f^{\left(1:n_{f}\right)}$ is the set of registration variables $\left\{f^{\left(i\right)},i=1,2,\cdots \;,n_{f}\right\}$ and *B*(·) is binomial distribution.

(26) $$\begin{array}{l} B\left(x,n,p\right)=\frac{n!}{x!\left(n-x\right)!}p^{x}{\left(1-p\right)}^{n-x} \end{array}$$

Therefore, the probability of image registration is obtained:

(27) $$\begin{array}{l} P\left(m=1|f^{\left(1:n_{f}\right)}\right)=\frac{P\left(f^{\left(1:n_{f}\right)}|m=1\right)P\left(m=1\right)}{P\left(f^{\left(1:n_{f}\right)}\right)}\\ \;=\frac{1}{1+\frac{P\left(f^{\left(1:n_{f}\right)}|m=0\right)P\left(m=0\right)}{P\left(f^{\left(1:n_{f}\right)}|m=1\right)P\left(m=1\right)}} \end{array}$$

In this case of $P\left(m=1|f^{\left(1:n_{f}\right)}\right)>P_{\min}$, the image is correctly registered. That is, if $B\left(n_{i},n_{f},p_{1}\right)P\left(m=1\right)>\frac{1}{\frac{1}{P_{min}}-1}$, then accept this point as the interior point, otherwise refuse.

According to the experience, we can get *P*(*m* = 1) = 10^−6^, *P*_*min*_ = 0.999. To make the image registration correct, we need to meet the following requirements:

(28) $$\begin{array}{l} n_{i}>\alpha +\beta n_{f}\;\left(\alpha =8.0,\beta =0.3\right) \end{array}$$

The mainly process of image alignment is to calculate the transformation parameter matrix model. The parameter matrix has been obtained in eliminating mismatched points by the RANSAC algorithm. Here is a detailed description of the process.

Projection transformation is usually used in image registration:

(29) $$\begin{array}{l} H=\left[\begin{array}{ccc} h_{11} & h_{12} & h_{13}\\ h_{21} & h_{22} & h_{23}\\ h_{31} & h_{32} & 1 \end{array}\right] \end{array}$$

Where the degree of freedom of *H* is 8. If *X*(*x*′, *y*′) and *X*(*x, y*) is a pair of registration points, then through projection transformation, there are:

(30) $$\begin{array}{l} \left(\begin{array}{c} x^{{}^{\prime }}\\ y^{{}^{\prime }}\\ w \end{array}\right)=\left(\begin{array}{ccc} h_{11} & h_{12} & h_{13}\\ h_{21} & h_{22} & h_{23}\\ h_{31} & h_{32} & 1 \end{array}\right)\left(\begin{array}{c} x\\ y\\ 1 \end{array}\right) \end{array}$$

The matrix is abbreviated as:

(31) $$\begin{array}{l} X^{{}^{\prime }}=HX \end{array}$$

Then, after the keypoints (*X*) in an image are transformed through the transformation matrix *H* into the matching image *X*′, Equations (25) and (26) can also be expressed as follows.

(32) $$\begin{array}{l} x^{{}^{\prime }}=\frac{h_{11}x+h_{12}y+h_{13}}{h_{31}x+h_{32}y+1} \end{array}$$

(33) $$\begin{array}{l} y^{{}^{\prime }}=\frac{h_{21}x+h_{22}y+h_{23}}{h_{31}x+h_{32}y+1} \end{array}$$

Since eight equations can calculate eight parameters. Only four pairs of registration points that are not collinear are needed to solve the parameters of the projection matrix, then Equation (30) can be written as follows:

(34) $$\begin{array}{l} \left[\begin{array}{cccccccc} x_{1} & y_{1} & 1 & 0 & 0 & 0 & -{x_{1}}^{\prime }x_{1} & -{x_{1}}^{\prime }y_{1}\\ 0 & 0 & 0 & x_{1} & y_{1} & 1 & -{y_{1}}^{\prime }x_{1} & -{y_{1}}^{\prime }y_{1}\\ x_{2} & y_{2} & 1 & 0 & 0 & 0 & -{x_{2}}^{\prime }x_{2} & -{x_{2}}^{\prime }y_{2}\\ 0 & 0 & 0 & x_{2} & y_{2} & 1 & -{y_{2}}^{\prime }x_{2} & -{y_{2}}^{\prime }y_{2}\\ \vdots & \vdots & \vdots & \vdots & \vdots & \vdots & \vdots & \vdots \\ \vdots & \vdots & \vdots & \vdots & \vdots & \vdots & \vdots & \vdots \\ x_{n} & y_{n} & 1 & 0 & 0 & 0 & -{x_{n}}^{\prime }x_{n} & -{x_{n}}^{\prime }y_{n}\\ 0 & 0 & 0 & x_{n} & y_{n} & 1 & -{y_{n}}^{\prime }x_{n} & -{y_{n}}^{\prime }y_{n} \end{array}\right]\left[\begin{array}{c} h_{11}\\ h_{12}\\ h_{13}\\ h_{21}\\ h_{22}\\ h_{23}\\ h_{31}\\ h_{32} \end{array}\right]=\left[\begin{array}{c} {x_{1}}^{\prime }\\ {y_{1}}^{\prime }\\ {x_{2}}^{\prime }\\ {y_{2}}^{\prime }\\ \vdots \\ \vdots \\ {x_{n}}^{\prime }\\ {y_{n}}^{\prime } \end{array}\right] \end{array}$$

Suppose the image gets the correct registration relationship. In that case, the number of internal points *n* is generally much larger than 4, so to ensure the best matrix parameters, the 2*n* system equations are often used.

When calculating the specific parameters of the projection matrix, it is necessary to minimize the back-projection error of interior points.

Let


$$\begin{array}{l} X=\left[\begin{array}{cccccccc} x_{1} & y_{1} & 1 & 0 & 0 & 0 & -{x_{1}}^{\prime }x_{1} & -{x_{1}}^{\prime }y_{1}\\ 0 & 0 & 0 & x_{1} & y_{1} & 1 & -{y_{1}}^{\prime }x_{1} & -{y_{1}}^{\prime }y_{1}\\ x_{2} & y_{2} & 1 & 0 & 0 & 0 & -{x_{2}}^{\prime }x_{2} & -{x_{2}}^{\prime }y_{2}\\ 0 & 0 & 0 & x_{2} & y_{2} & 1 & -{y_{2}}^{\prime }x_{2} & -{y_{2}}^{\prime }y_{2}\\ \vdots & \vdots & \vdots & \vdots & \vdots & \vdots & \vdots & \vdots \\ \vdots & \vdots & \vdots & \vdots & \vdots & \vdots & \vdots & \vdots \\ x_{n} & y_{n} & 1 & 0 & 0 & 0 & -{x_{n}}^{\prime }x_{n} & -{x_{n}}^{\prime }y_{n}\\ 0 & 0 & 0 & x_{n} & y_{n} & 1 & -{y_{n}}^{\prime }x_{n} & -{y_{n}}^{\prime }y_{n} \end{array}\right],h=\left[\begin{array}{c} h_{11}\\ h_{12}\\ \vdots \\ h_{32} \end{array}\right],y=\left[\begin{array}{c} {x_{1}}^{\prime }\\ {y_{1}}^{\prime }\\ \vdots \\ {x_{n}}^{\prime }\\ {y_{n}}^{\prime } \end{array}\right] \end{array}$$

The 2*n* ∗ 8 matrix form of the original equations is *X* · *h* = *y*.

The error of back projection is as follows

(35) $$\begin{array}{l} E=\sum_{i=1}^{n}{\left({x_{i}}^{{}^{\prime }}-x_{i}\right)}^{2}+{\left({y_{i}}^{{}^{\prime }}-y_{i}\right)}^{2} \end{array}$$

In combination with Equations (28) and (29):

(36) $$\begin{array}{l} E=\sum_{i=1}^{n}{\left({x^{{}^{\prime }}}_{i}-\frac{h_{11}x_{i}+h_{12}y_{i}+h_{1s}}{h_{s1}x_{i}+h_{32}y_{i}+1}\right)}^{2}+{\left({y_{i}}^{{}^{\prime }}-\frac{h_{21}x_{i}+h_{22}y_{i}+h_{2s}}{h_{s1}x_{i}+h_{s2}y_{i}+1}\right)}^{2} \end{array}$$

It is also equivalent to:

(37) $$\begin{array}{l} S\left(h\right)=||Xh-y|{|}^{2} \end{array}$$

The solution of the objective function is the solution of an interior point matching pair with the minimum reverse error.

Then it is concluded that when *h* = *h* the minimum value is *S*(*h*),

(38) $$\begin{array}{l} h=argmin\left(S\left(h\right)\right) \end{array}$$

The differential solution is as follows:

(39) $$\begin{array}{l} h={\left(X^{T}X\right)}^{-1}X^{T}y \end{array}$$

### Endoscopic Image Fusion Results

After obtaining the optimal transformation matrix between the images to be spliced, that is, after the image registration is completed, image fusion needs to be used to merge the two images into one image. However, due to uneven illumination during shooting, the brightness of the overlapping parts of the two images is very different. Or the image is deformed due to lens distortion. These eventually lead to significant gaps in the stitched image, which people generally call ghosts. In order to achieve the consistency of human vision, we need to remove this trace. This paper adopts the weighted fusion algorithm of gradual-in and gradual-out, which can make the image transition smoothly and avoid obvious boundary problems.

Suppose that the two images *I*_1_ and *I*_2_ are needed to be spliced, and *I* is the fused image:

(40) $$\begin{array}{l} I\left(x,y\right)=\{\begin{array}{c} I_{1}\left(x,y\right)\\ \frac{\left(d_{1}I_{1}\left(x,y\right)+d_{2}I_{2}\left(x,y\right)\right)}{2}\\ I_{2}\left(x,y\right) \end{array}\begin{array}{c} \left(x,y\right)\in I_{1}\\ \;\left(x,y\right)\in \left(I_{1}\cap I_{2}\right)\\ \left(x,y\right)\in I_{2} \end{array} \end{array}$$

Where, *d*_1_ and *d*_2_ represent the weight values, and they are related to the width of the coincidence part, i.e., $d_{1}=\frac{1}{width}$, where *width* is the width of the coincidence part. *d*_1_ and *d*_2_ must also satisfy the following requirements: *d*_1_ + *d*_2_ = 1,0 < *d*_1_ < 1,0 < *d*_2_ < 1. In the process of fusion, the image *d*_1_ is gradually changed from 1 to 0 and *d*_2_ is gradually changed from 0 to 1 in the overlapped part. Then, the image is slowly and smoothly transitioned from *I*_1_ to *I*_2_ in the overlapping part.

## Experimental Results

The experimental materials used in the experiment are two sets of three-dimensional images of the heart model and its corresponding CT scan data and a set of actual three-dimensional images of the soft tissue of the heart provided by Imperial College London, which can be found on the public data website http://hamlyn.doc.ic.ac.uk/vision/.

Two groups of experiments are carried out to verify the accuracy of the improved algorithm proposed in this paper. And the unpurified matching pair and directly use the RANSAC purification algorithm are as comparison.

**Figure 2:**
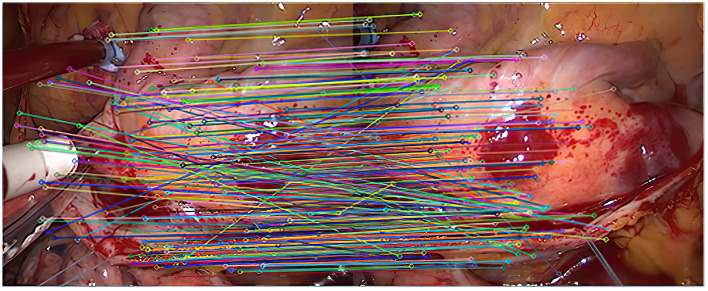
The first matching result of feature points.

**Figure 3:**
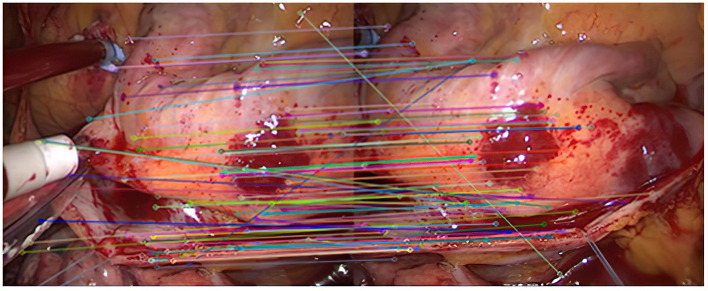
Results of initial screening of matching pairs.

**Figure 4:**
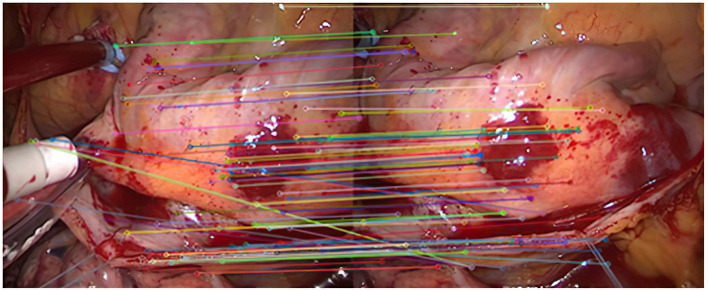
The result after removing the mismatch.

Experiment 1: 50 groups of registration experiments were carried out on the images in endoscope image set (I), and the effect Figures 2–**4** were shown, and 8 groups of data in the experiment were randomly counted, as shown in Table 1.

**Table 1:** Comparison of purification algorithms for feature point matching.

** 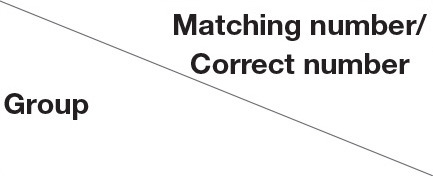 **	**Matching algorithm**		
	**Before purification**	**RANSAC purification**	**Improved purification algorithm**
1	500/411	305/280	242/234
2	471/377	310/283	253/248
3	446/355	321/290	252/246
4	500/417	285/255	258/251
5	435/346	310/279	249/240
6	452/365	298/265	240/233
7	489/391	293/261	250/242
8	493/398	317/280	245/235
Average value	80.7%	89.9%	96.9%

As shown in Figure 2, it is the result of the initial matching of the images in the endoscopic image set (I) using the Euclidean distance. The two ends of the line in the figure are the matching feature points of the two images to be registered. It can be seen that the number of feature points is relatively dense. This article uses lines with different colors to connect the key points in the image to make them easier to distinguish. It can be seen from the figure that there are more mismatched points because the lines connecting the feature points are not very neat. This is because there are more similar areas in the adjacent endoscopic images.

After the initial screening of the registration points using K nearest neighbors, 127 pairs are obtained, as shown in Figure 3.

As shown in Figure 4, there are 113 registration pairs purified by the RANSAC algorithm.

**Figure 5:**
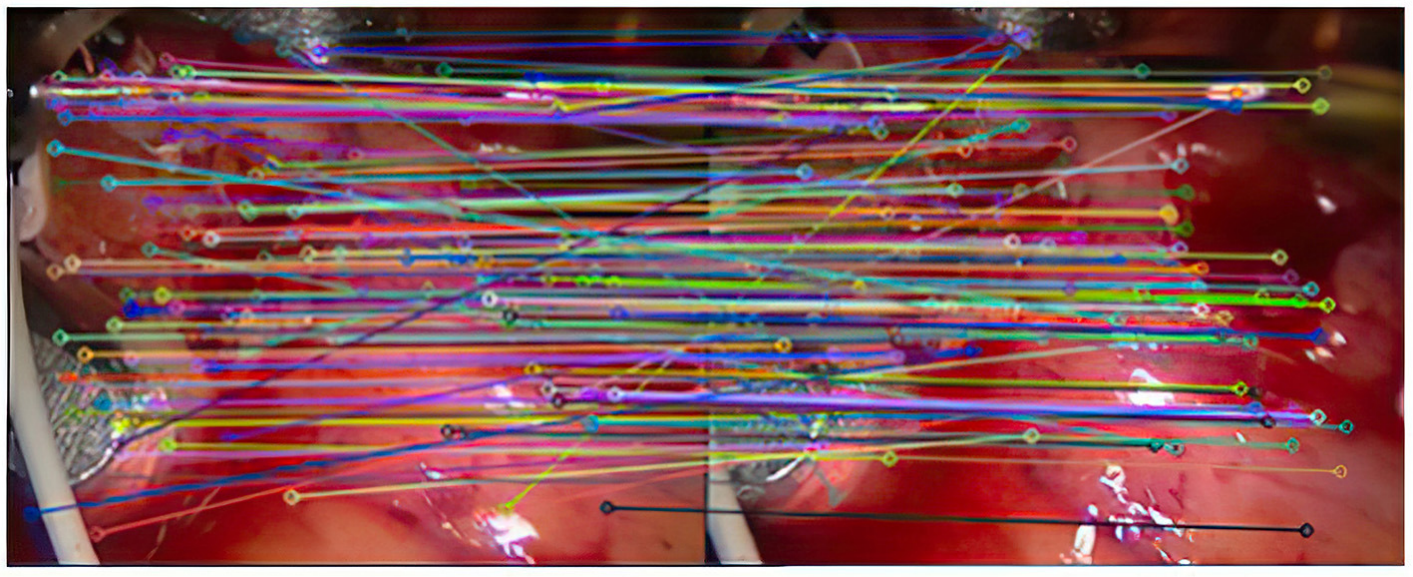
Initial matching results of feature points.

**Figure 6:**
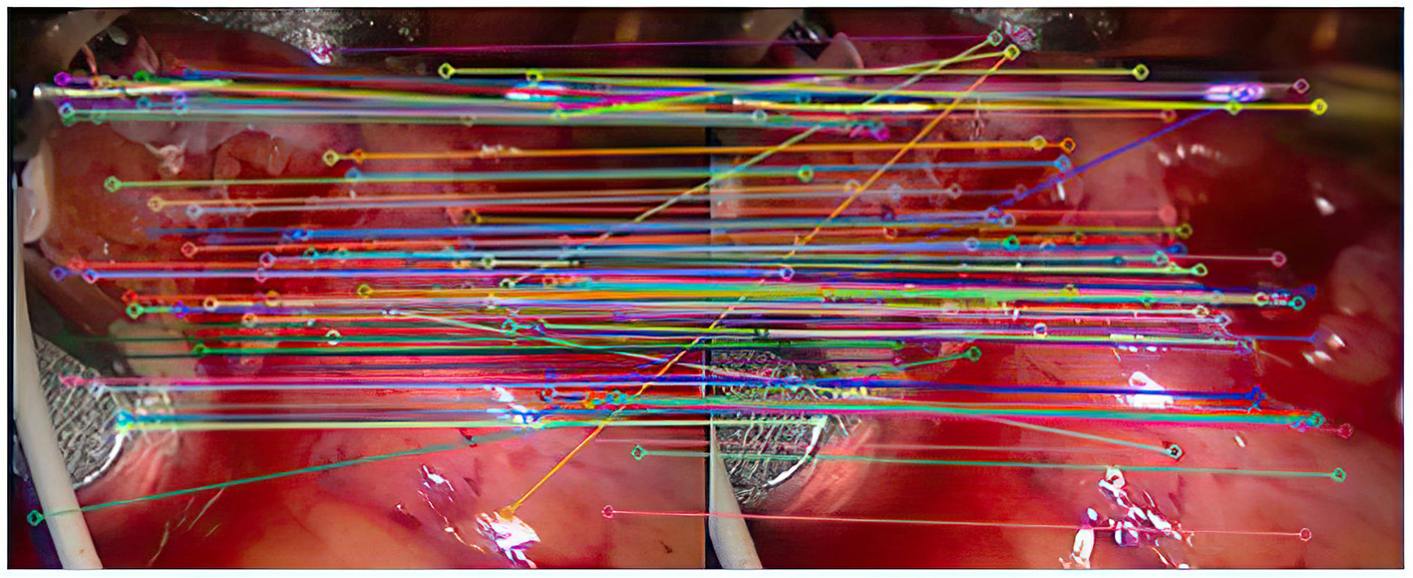
Result of RANSAC after removing mismatches.

**Figure 7:**
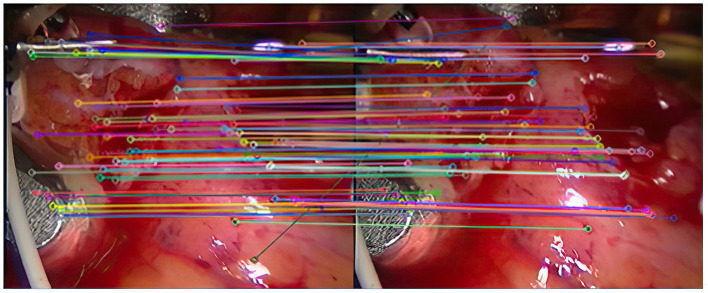
The result of eliminating mismatches by improved purification algorithm.

Experiment 2: Many registration experiments have been carried out on the images in the endoscopic image set (II) to verify the improved algorithm further. The results are shown in Figures 5–7. Figure 5 is the initial matching results of feature points, and Figure 6 is the results after removing mismatches by RANSAC directly. Figure 7 is the results after removing mismatches by using the improved purification algorithm. Similarly, eight groups of data in the experiment were randomly counted, as shown in Table 2.

**Table 2:** Comparison of purification algorithms for feature point matching.

** 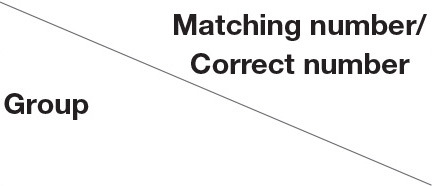 **	**Matching algorithm**		
	**Before purification**	**RANSAC purification**	**Improved purification algorithm**
1	305/244	153/137	98/94
2	308/249	151/133	100/97
3	312/251	156/139	103/99
4	300/241	147/131	95/91
5	315/253	158/142	107/104
6	299/239	149/134	99/95
7	303/242	152/135	102/99
8	307/248	151/132	101/96
Average value	80.3%	89.0%	96.2%

**Figure 8:**
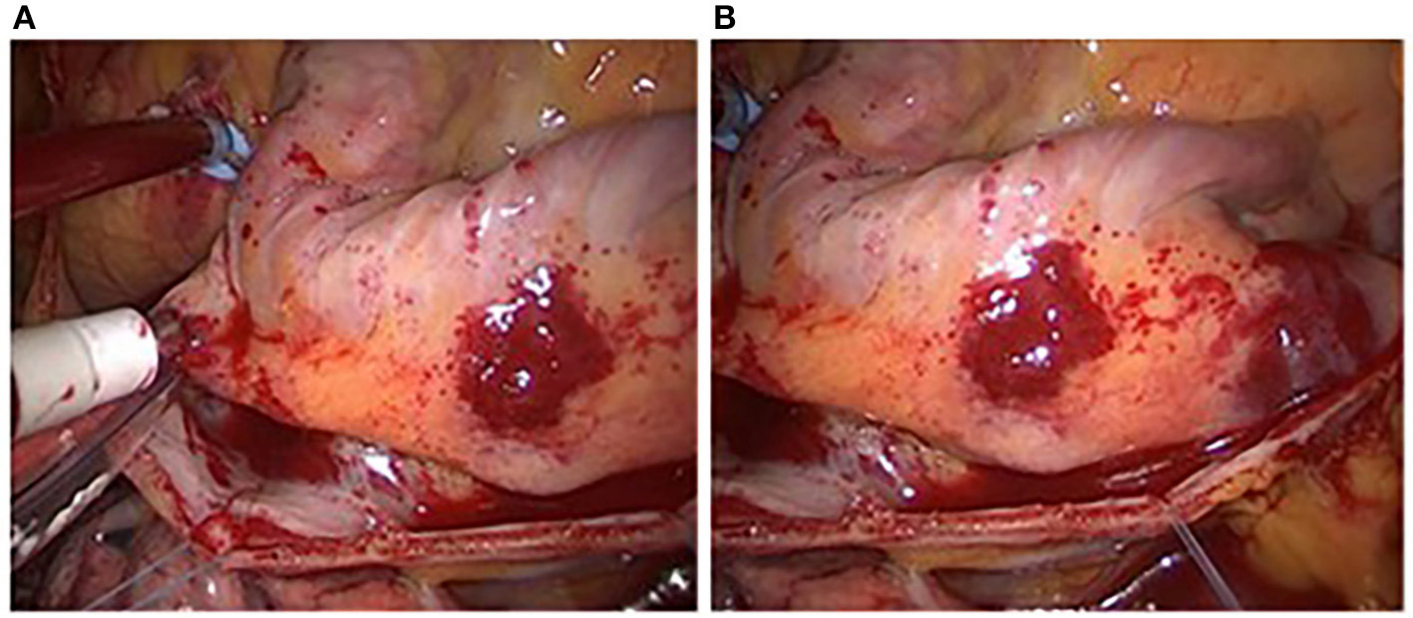
Two endoscopic images to be spliced. **(A)** Reference image; **(B)** image to be registered.

**Figure 9:**
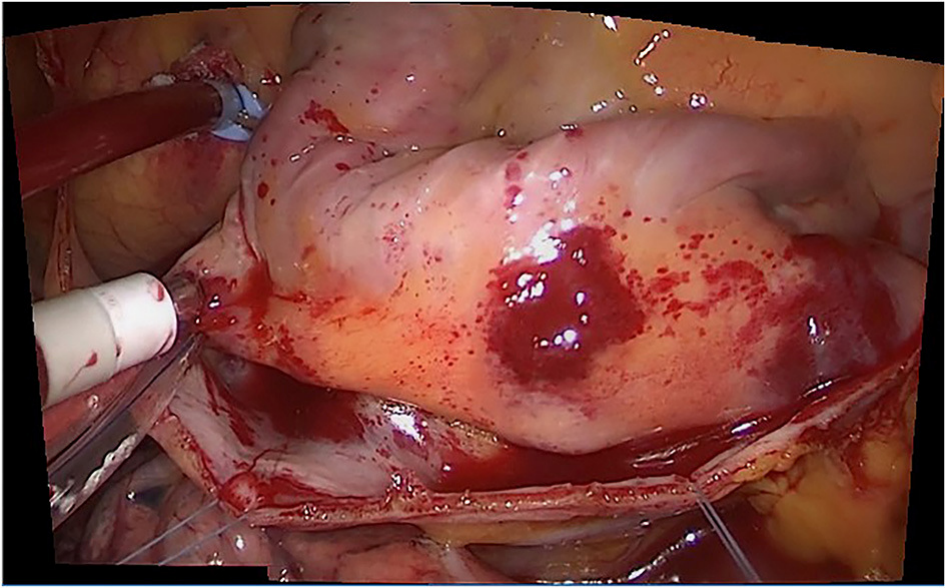
The result of the improved SIFT algorithm.

Figure 8 shows two adjacent endoscopic images with overlapping areas called reference image and registration image. Their sizes are both 500 × 412. Figure 9 is the result of the fusion based on the Improved SIFT image mosaic algorithm.

## Discussion

In this paper, the image Mosaic method based on SIF is used to combine the two commonly used methods in feature point purification. The experimental results show that the matching of feature points is greatly improved. In addition, because there is no complex structure such as neural network (Hang et al., 2020), the method in this paper has the characteristics of simple structure and short running time. Discussion

This experiment focuses on the endoscopic image mosaic technology based on improved SIFT. After the experiment, we can draw the following conclusions.

1. As can be seen from Figure 3, after completing the initial screening, the number of mismatching points is greatly reduced. Although there are still some mismatching point pairs, the accuracy rate has been significantly improved, and the workload of RANSAC is greatly reduced. Comparing Figure 3, we can see that the number of mismatching points in Figure 4 is significantly reduced, and the matching lines are also neat. It means that RANSAC algorithm is effective.
2. It can be seen from Figures 6, 7 that the mosaic effect is relatively good. There is no obvious splicing seam, and the image transition is more natural. The experiment uses C + + and OpenCV library to simulate on vs. 2013. The algorithm's running time is 3,145 Ms. The algorithm is time-consuming and computationally heavy, which cannot meet the real-time requirements of endoscopic image mosaic.
3. From Tables 1, 2, we can see many mismatches in the feature matching pairs obtained based on Euclidean distance. After using the RANSAC purification algorithm directly, the matching accuracy has been improved. However, there are still mismatching pairs, which will greatly impact the subsequent image mosaic. After the improved purification algorithm, the mismatch pairs are eliminated, which is better than using RANSAC directly. The RANSAC purification algorithm is improved by about 7 percentage points, which provides reliable data for subsequent applications, showing that the improved purification algorithm in this paper is effective for eliminating mismatches.

Experts and scholars have done a lot of research work in image Mosaic. However, due to the particularity of minimally invasive surgery environment, usually insufficient light and soft tissue deformation, endoscope imaging is often disturbed greatly (Luo et al., 2020). Therefore, there are not many researches on endoscope image Mosaic technology in China. The key problems of minimally invasive surgery endoscope image Mosaic are still robustness and real-time. Although this paper improves the robustness of endoscope image Mosaic to a certain extent and achieves certain results, there are still many problems to be studied and solved, mainly including:

1. The transformation matrix with 8 degrees of freedom is adopted in image registration in this paper. The complex transformation model can be further studied to obtain higher registration accuracy. In this paper, image registration is local registration, that is to solve the transform relationship between the two images, image stitching also need to consider the transformation between the two images, but this can lead to cumulative error, caused the splicing image fuzzy, therefore, the next step to research global registration, reduce the cumulative error, improve accuracy of registration, further improve the effect of stitching images.
2. This paper is based on still images, but the future research on dynamic images is also essential. Combining video processing with image Mosaic technology, the panoramic image obtained not only has wide field of view, high resolution panoramic image, but also contains dynamic elements in the image. In addition, the image Mosaic done in this paper is two-dimensional, which can be extended to three-dimensional image Mosaic in the future to obtain more intuitive endoscope images.
3. Since it is difficult to accurately evaluate the effect of spliced images, this paper chooses the artificial observation method to evaluate the effect of images. In the future, some criteria for accurate evaluation of images can be proposed, and then experimental verification can be carried out, so that the effect of spliced images can be evaluated more objectively and conveniently.

## Conclusion

Because there are many similar regions in endoscopic images, there are many matching errors, which will affect the final stitching effect. An improved pair purification algorithm is proposed to solve the problem. Firstly, the feature point matching algorithm based on K-nearest neighbor bidirectional matching is used for rough registration. Then RANSAC is used to complete fine registration. In this way, the mismatching rate is greatly reduced by combining the two methods. Then, according to the exact matching point pairs, the image matching relationship is determined. The image transformation matrix is estimated. Finally, a gradual in and out fusion is used to complete endoscopic images' seamless stitching. The experimental results show that the image's effect is still good. However, because of the stitching algorithm's complexity, the stitching time is too long, which does not meet the real-time requirements of endoscopic image mosaic. Finally, several experiments are carried out to verify the performance of the improved feature pair purification algorithm. The experimental results show that the matching rate of feature points is greatly improved, proving the algorithm's effectiveness.

## Data Availability Statement

The original contributions presented in the study are publicly available. This data can be found here: https://imperialcollegelondon.app.box.com/s/kits2r3uha3fn7zkoyuiikjm1gjnyle3.

## Author Contributions

SL, BY, WZ, and LY contributed to the design of this work. YL contributed to the writing of the manuscript. JT and YL designed the model, implemented it in the framework, together with WZ. LY revised the manuscript. All authors listed have made substantial, direct, and intellectual contribution to the work they have also approved it for publication.

## Funding

This work was jointly supported by the Sichuan Science and Technology Program (2021YFQ0003).

## Conflict of Interest

The authors declare that the research was conducted in the absence of any commercial or financial relationships that could be construed as a potential conflict of interest.

## Publisher's Note

All claims expressed in this article are solely those of the authors and do not necessarily represent those of their affiliated organizations, or those of the publisher, the editors and the reviewers. Any product that may be evaluated in this article, or claim that may be made by its manufacturer, is not guaranteed or endorsed by the publisher.
